# Oxidative Stress and Cirrhosis Severity: A Retrospective Cohort Analysis of Predictive and Interactive Effects with Inflammation

**DOI:** 10.3390/metabo15110711

**Published:** 2025-10-30

**Authors:** Vlad Pădureanu, Lidia Boldeanu, Denisa Floriana Vasilica Pîrșcoveanu, Dalia Dop, Ramona Cioboată, Anca Bobîrcă, Virginia Maria Rădulescu

**Affiliations:** 1 Department of Internal Medicine, University of Medicine and Pharmacy of Craiova, 200349 Craiova, Romania; vlad.padureanu@umfcv.ro; 2 Department of Microbiology, University of Medicine and Pharmacy of Craiova, 200349 Craiova, Romania; lidia.boldeanu@umfcv.ro; 3 Department of Neurology, University of Medicine and Pharmacy Craiova, 200349 Craiova, Romania; 4 Department of Pediatrics, University of Medicine and Pharmacy Craiova, 200349 Craiova, Romania; 5 Department of Pneumology, University of Medicine and Pharmacy Craiova, 200349 Craiova, Romania; ramona.cioboata@umfcv.ro; 6 Department of Internal Medicine and Rheumatology, ‘Carol Davila’ University of Medicine and Pharmacy, 020021 Bucharest, Romania; anca.bobirca@umfcd.ro; 7 Department of Medical Informatics and Biostatistics, University of Medicine and Pharmacy of Craiova, 200349 Craiova, Romania; virginia.radulescu@umfcv.ro

**Keywords:** oxidative stress, liver cirrhosis, inflammation, albumin, INR

## Abstract

Background/Objectives: Oxidative stress is a central mechanism in the pathogenesis of cirrhosis, yet its clinical significance relative to established predictors remains unclear. Methods: We conducted a retrospective cohort study of 90 patients with cirrhosis hospitalized between October 2024 and March 2025. Clinical data, biochemical parameters, systemic inflammatory indices, and oxidative stress markers [malondialdehyde (MDA), 8-epi-prostaglandin F2α (8-epi-PGF2α)] were assessed at admission. Statistical analyses included non-parametric group comparisons, Spearman correlations, logistic regression with interaction terms, ROC analysis with bootstrap confidence intervals, model calibration and discrimination metrics, reclassification indices (NRI, IDI), and decision curve analysis (DCA). Results: Patients with advanced encephalopathy (HE3) had significantly higher MDA levels compared with HE1 (123.4 [107.6–248.4] vs. 131.0 [66.9–301.1] ng/mL; *p* = 0.021), while 8-epi-PGF2α showed a non-significant but consistent trend. Both oxidative markers correlated with biochemical dysfunction (MDA with INR and albumin; 8-epi-PGF2α with direct bilirubin). ROC analyses demonstrated modest discriminative ability (AUC 0.55–0.60) compared with albumin (AUC 0.74–0.90) and INR (AUC 0.72–0.88). In regression models, albumin remained the strongest independent predictor, whereas oxidative markers did not retain significance. Interaction models suggested that oxidative stress exerted context-dependent effects, particularly in patients with elevated inflammatory indices. Incremental predictive value beyond age and albumin was minimal (ΔAUC ≤ 0.01; NRI + 2–4%). DCA confirmed no added clinical utility. Conclusions: Classical clinical markers, particularly albumin and INR, dominate predictive accuracy in cirrhosis. Oxidative stress markers lack independent predictive power but consistently associate with worsening encephalopathy and liver dysfunction, underscoring their biological relevance and suggesting their role is best understood in conjunction with systemic inflammation.

## 1. Introduction

Hepatic dysfunction results from liver cirrhosis, a serious worldwide health concern marked by the progressive replacement of liver tissue with fibrosis and regenerating nodules. Oxidative stress, a disorder resulting from an imbalance between the generation of reactive oxygen species (ROS) and the antioxidant defenses of the cell, is one of the fundamental mechanisms driving the pathophysiology of liver cirrhosis. One important element in the development of liver cirrhosis is known to be oxidative stress. It amplifies inflammatory signaling and fibrogenesis in the liver by harming cellular constituents such as proteins, lipids, and DNA [1].

Liver fibrosis and cirrhosis are caused and progress by a variety of cell types, cytokines, and microRNAs. All cases of liver cirrhosis share certain pathological characteristics, such as hepatocyte necrosis and degeneration, fibrotic tissues, regenerating nodules replacing the liver parenchyma, and loss of liver function. Oxidative stress and inflammation are two related processes that cause structural and functional changes in the liver when it is exposed to high levels of ethanol [2]. As liver fibrosis progresses, ethanol may enhance the generation of reactive oxygen and nitrogen species (ROS, RNS), which can trigger pro-fibrogenic cytokines, the release of several inflammatory markers, and the synthesis of collagen [3,4]. ROS are molecules that contain oxygen and are created during regular metabolism. The body has both enzymatic and non-enzymatic antioxidant mechanisms that can counteract the negative effects of endogenous ROS [5]. To neutralize the free radicals produced by viruses and other endogenous and foreign substances that the liver processes, the liver typically maintains a balance between internal antioxidants and ROS. Under state. In certain conditions, an increase in ROS generation or antioxidant depletion can shift the oxidative to antioxidative balance in favor of the oxidative state.

By altering the primary biological molecules (DNA, proteins, and lipids), oxidative stress leads to liver damage [6]. According to earlier research, lipid peroxidation products, DNA, and protein oxidation all play a role in the development and progression of liver fibrosis by influencing signaling pathways linked to gene transcription, protein expression, apoptosis, and hepatic stellate cell activation [7,8]. Inflammation is a crucial immune response event where inflammatory cells invade the body to combat various aggressive stimuli.

Researchers have long been interested in the strong relationship between inflammation and oxidative stress in the development of liver disease. In addition to producing more ROS and RNS, excessive inflammatory cells can also raise the expression of genes that code for proinflammatory cytokines. Most people agree that inflammation and oxidative stress are closely associated and form a vicious cycle that contributes to the development of cirrhosis and, eventually, hepatocellular carcinoma in liver disorders [9].

The importance of hematological markers of inflammation from the complete blood count (CBC) panel, such as neutrophil/lymphocyte (NLR), monocyte/lymphocyte (MLR), and platelet/lymphocyte (PLR) ratios, systemic immune-inflammation index (SII), systemic inflammation response index (SIRI) in determining the prognosis of different illnesses has been the focus of recent research trends [10,11,12]. As prognostic indicators for cancer, sepsis, heart disease, pneumonia, and acute respiratory distress syndrome, NLR and PLR have therefore been verified [13,14,15]. The function of these ratios as predictive indicators of disease outcome in liver cirrhosis patients has not been thoroughly examined in research. To the best of our knowledge, none of these have discussed using these indices to evaluate the relationship between inflammation, oxidative stress, and the severity of liver disease.

Notably, oxidative stress and antioxidant defenses may differ by sex: estrogens have been associated with enhanced antioxidant enzyme activity (e.g., superoxide dismutase (SOD), glutathione peroxidase (GPX)), whereas men may display higher nicotinamide adenine dinucleotide phosphate oxidase (NADPH-oxidase) activity and a more intense inflammatory tone. These biological gradients motivate reporting sex distribution and a cautious, sex-aware interpretation of oxidative biomarkers in cirrhosis [16,17,18].

This study explicitly probes interactions between oxidative stress and systemic inflammatory indices (e.g., MDA × NLR; 8-epi-PGF2α × SII) and evaluates the incremental performance of such markers over clinical baselines using an integrated set of metrics (receiver operating characteristic (ROC), net reclassification improvement/integrated discrimination improvement (NRI/IDI), and decision curve analysis (DCA)). This design clarifies not only whether oxidative biomarkers correlate with severity, but whether—and to what extent—they provide actionable prognostic value beyond standard clinical variables.

To develop new predictive tools for a non-invasive paraclinical investigation of disease outcomes in patients with liver cirrhosis, the current study aimed to ascertain the utility of hematological indicators in evaluating the relationship between inflammation and oxidative stress.

## 2. Materials and Methods

### 2.1. Study Design and Population

This was a single-center retrospective cohort study conducted between October 2024 and March 2025 on a cohort of 90 patients diagnosed with liver cirrhosis and hospitalized at the 2nd Medical Clinic of the Craiova County Emergency Hospital. Cirrhosis was defined based on a combination of clinical, laboratory, and imaging criteria in accordance with established guidelines.

Inclusion criteria were: (i) age ≥ 18 years; (ii) confirmed diagnosis of cirrhosis; and (iii) availability of complete clinical and laboratory data at admission. Exclusion criteria were: (i) diagnosis of hepatocellular carcinoma or other malignancies; (ii) concomitant acute infections at the time of admission; (iii) advanced chronic kidney disease requiring dialysis; and (iv) incomplete datasets.

The study protocol complied with the ethical standards of the Declaration of Helsinki and was approved by the Institutional Ethics Committee of the University of Medicine and Pharmacy of Craiova. Informed consent was obtained from all patients or their legal representatives at the time of hospitalization.

### 2.2. Clinical and Laboratory Data

Demographic data (age, sex) and clinical variables (days of hospitalization, presence and grade of hepatic encephalopathy, and presence and degree of ascites) were collected at the time of admission. Hepatic encephalopathy was graded according to the West Haven criteria (grades 1–3), while ascites was classified as mild, moderate, or large based on clinical evaluation.

Laboratory data were obtained from venous blood samples collected at admission and analyzed according to institutional protocols. The following biochemical parameters were assessed: serum albumin (g/dL), international normalized ratio (INR), total bilirubin and direct bilirubin (mg/dL), and serum creatinine (mg/dL). All assays were performed using standardized hospital laboratory techniques with internal quality control.

In addition, routine hematological parameters (hemoglobin, white blood cell count, neutrophils, lymphocytes, monocytes, and platelets) were recorded and used for the derivation of systemic inflammatory indices.

### 2.3. Definitions of Variables and Indices

The severity of hepatic encephalopathy (HE) was classified according to the West Haven criteria, with grades 1 to 3 indicating progressive neurological impairment. Overall liver disease severity was evaluated using the Child-Pugh classification, which integrates serum bilirubin, serum albumin, INR, ascites, and encephalopathy. Patients in the present cohort were categorized into Child-Pugh classes B and C.

Inflammatory indices were calculated from routine hematological parameters:

- NLR = neutrophil count/lymphocyte count.
- PLR = platelet count/lymphocyte count.
- MLR = monocyte count/lymphocyte count.
- SII = (platelet count × neutrophil count)/lymphocyte count.
- SIRI = (neutrophil count × monocyte count)/lymphocyte count.
- Oxidative stress markers included:
- Malondialdehyde (MDA), measured as a byproduct of lipid peroxidation and expressed in ng/mL.
- 8-epi-prostaglandin F2α (8-epi-PGF2α), a stable F2-isoprostane reflecting oxidative stress–induced lipid peroxidation, expressed in pg/mL.

All indices and biomarkers were analyzed as continuous variables. For exploratory analyses, tertiles of MDA and 8-epi-PGF2α were also derived and used in trend tests.

### 2.4. Measurement of Oxidative Stress Markers

Oxidative stress markers were determined from venous blood samples collected at admission, before the initiation of specific therapy. Serum was separated by centrifugation and stored at –80 °C until analysis.

For the quantitative measurement of serum concentrations of MDA and 8-epi-PGF2α, the Enzyme-Linked Immunosorbent Assay (ELISA) technique was used at the Immunology Laboratory of the University of Medicine and Pharmacy of Craiova.

#### 2.4.1. Assays and Specificity

We utilized commercially available ELISA kits from Elabscience (Houston, TX, USA):

- MDA (Catalog No: E-EL-0060; sensitivity: 18.75 ng/mL; detection range: 31.25–2000 ng/mL);
- 8-epi-PGF2α (Catalog No: E-EL-0041; sensitivity: 9.38 pg/mL; detection range: 15.63–1000 pg/mL).

Based on the manufacturer’s Instructions for Use (IFU) and lot-specific validation sheets, all antibodies used in these assays were generated against hapten-conjugated targets and evaluated for cross-reactivity with structurally related MDA and 8-epi-PGF2α. The cross-reactivity data for each analyte indicate no significant cross-reactivity or interference between Universal 8-epi-PGF2α (https://789.bio/ea/OC4Om5, accessed on 21 September 2025), Universal MDA (https://789.bio/ea/GG8Gy9, accessed on 21 September 2025), and their analogs.

Performance: Precision—intra-assay precision (precision within an assay): 4.07–6.86 CV%; inter-assay precision (precision between assays): 7.09–7.77 CV%.

#### 2.4.2. Pre-Analytical Handling

To ensure analyte stability and minimize the risk of non-specific oxidation or degradation, all samples were processed within 30 min of collection. After clotting and centrifugation, serum was divided into cryovials and stored at −80 °C. Each sample was thawed only once before analysis. When applicable, manufacturer-provided diluents with stabilizing agents were used to reduce assay interference.

#### 2.4.3. Analytical Limitations and Quality Controls

In-house spike-and-recovery or parallelism experiments were not conducted. Therefore, although manufacturer documentation states high specificity and no significant cross-reactivity, analytical interference cannot be completely ruled out.

#### 2.4.4. Test Principle

This ELISA kit operates on the competitive ELISA principle (Elabscience, Houston, TX, USA). The included micro-ELISA plate is pre-coated with Universal MDA and 8-epi-PGF2α. During the test, Universal MDA and 8-epi-PGF2α in samples or standards compete with a fixed amount of the same on the plate for binding to biotinylated detection antibodies specific to Universal MDA and 8-epi-PGF2α. Excess conjugate, along with any unbound samples or standards, is washed away. Horseradish peroxidase (HRP)-conjugated avidin is then added to each well and incubated. Next, a TMB substrate solution is added. The reaction is stopped with a stop solution, and the resulting color change is measured spectrophotometrically at 450 ± 2 nm using the Asys Expert Plus UV G020 150 Microplate Reader from ASYS Hitech GmbH, Eugendorf, Austria. The concentration of Universal MDA and 8-epi-PGF2α in the samples is determined by comparing their optical density (OD) to the standard curve.

### 2.5. Statistical Analysis

Data were initially recorded and organized in Microsoft Excel (Microsoft Corp., Redmond, WA, USA), and statistical analyses were performed using SPSS version 26.0 (IBM Corp., Armonk, NY, USA). Continuous variables were tested for normality using the Shapiro–Wilk test. Given the non-Gaussian distribution of most parameters, results were expressed as medians with interquartile ranges (IQR). Categorical variables were presented as counts and percentages.

Group comparisons were performed using the Mann–Whitney U test (for two groups) and the Kruskal–Wallis test (for three or more groups), with post hoc pairwise Mann–Whitney U tests where applicable. Associations between continuous variables were assessed using Spearman’s rank correlation coefficient.

Binary logistic regression analyses were used to evaluate predictors of severe hepatic encephalopathy (grade 3 vs. grades 1–2) and of Child-Pugh class C vs. class B. Both simple models (age, albumin, MDA, 8-epi-PGF2α) and interaction models (MDA × NLR, 8-epi-PGF2α × SII) were tested. While oxidative stress markers alone did not emerge as independent predictors, their inclusion in interaction terms highlighted a context-dependent effect, with marginal effects illustrating stronger associations in patients with elevated inflammatory burden. Odds ratios (OR) with 95% confidence intervals (CI) were reported, and marginal effects were additionally computed for interaction models to aid interpretation.

Model performance was assessed using the area under the receiver operating characteristic curve (AUC), with 95% CI estimated by 2000 bootstrap resamples, as well as the Brier score and Hosmer–Lemeshow goodness-of-fit test. Clinical utility was evaluated through decision curve analysis (DCA). Incremental predictive value was assessed by net reclassification improvement (NRI) and integrated discrimination improvement (IDI), comparing a reference model (age + albumin) with extended models including oxidative stress markers.

For exploratory analyses, tertile categorization of oxidative stress markers was applied, and trend tests were performed. Sensitivity analyses were conducted using log-transformed skewed variables (MDA, 8-epi-PGF2α, SII, SIRI) and standardized covariates (z-scores); results were consistent with the primary findings. Sensitivity analyses were conducted using log-transformed skewed variables (MDA, 8-epi-PGF2α, SII, SIRI) and standardized covariates (z-scores); results were consistent with the primary findings. For interaction models, continuous predictors were standardized to z-scores (mean = 0, SD = 1), which are denoted in the tables with the prefix ‘z_’. Multicollinearity was assessed using variance inflation factors (VIF).

The *p*-value < 0.05 was considered statistically significant.

Continuous predictors were mean-centered before forming interaction terms. Analyses used complete-case datasets (no imputation); when events-per-variable (EPV) fell below conventional thresholds, models were explicitly labeled exploratory, and internal validation of discrimination used bootstrap confidence intervals.

Exploratory framing and model complexity. Given the modest sample size (*n* = 90), all multivariable analyses—including models with prespecified interaction terms—were treated as exploratory. We constrained model complexity a priori, avoided data-driven stepwise procedures, inspected collinearity (variance inflation factors), and reported effect sizes with 95% confidence intervals to emphasize estimation over dichotomous testing. Findings are interpreted with appropriate caution pending external validation.

## 3. Results

### 3.1. Baseline and Clinical Characteristics

A total of 90 patients with liver cirrhosis were included in the analysis. The median age was 58 years (IQR 50–66), and 54% of the cohort were male. In terms of disease severity, 62% of patients were classified as Child-Pugh class B and 38% as class C. HE was present in varying degrees: 41% of patients were classified as grade 1, 34% as grade 2, and 25% as grade 3.

The cohort included both women and men in approximately similar proportions. A brief sex-aware check of oxidative biomarkers (MDA and 8-epi-PGF2α) did not suggest material differences by sex; given limited power, formal sex-stratified inference was not pursued, and these observations do not alter the main conclusions.

Baseline characteristics stratified by Child-Pugh class are summarized in Table 1. Compared with patients in class B, those in class C had markedly lower serum albumin levels (2.50 [2.20–2.60] g/dL vs. 2.95 [2.73–3.40] g/dL, *p* < 0.001, Mann–Whitney U test), and significantly higher INR (1.60 [1.40–1.82] vs. 1.22 [1.16–1.41], *p* < 0.001), total bilirubin (4.80 [3.76–6.60] vs. 0.97 [0.63–2.02] mg/dL, *p* < 0.001), direct bilirubin (3.25 [2.30–4.30] vs. 0.43 [0.28–0.87] mg/dL, *p* < 0.001), and creatinine (1.30 [1.04–1.60] vs. 1.03 [0.95–1.21] mg/dL, *p* = 0.003). These differences are consistent with the metabolic decompensation and impaired synthetic capacity typically observed in advanced cirrhosis.

In contrast, markers of oxidative stress (MDA and 8-epi-PGF2α) and inflammatory indices (NLR and SII) did not reach statistical significance between Child-Pugh classes. However, both oxidative markers showed numerically higher values in the more advanced stage, suggesting a potential trend toward increased oxidative burden with progression of liver dysfunction, even if not independently discriminatory.

Baseline characteristics according to the severity of hepatic encephalopathy are detailed in Table 2. Albumin showed a stepwise decline across the spectrum of HE, from 3.00 [2.80–3.40] g/dL in HE1 to 2.60 [2.20–2.70] g/dL in HE2 and 2.50 [2.35–2.50] g/dL in HE3 (*p* < 0.001, Kruskal–Wallis test). In parallel, INR increased steadily (1.18 [1.14–1.41] in HE1, 1.53 [1.40–1.64] in HE2, and 1.60 [1.40–1.81] in HE3; *p* < 0.001), while total bilirubin rose from 0.98 [0.63–2.36] mg/dL in HE1 to 4.50 [2.40–5.73] in HE2 and 5.71 [3.90–7.78] in HE3 (*p* < 0.001). Direct bilirubin followed a similar gradient (0.46 [0.28–1.23] vs. 2.60 [1.73–3.35] vs. 3.30 [2.35–5.00] mg/dL; *p* < 0.001).

Creatinine levels were also significantly higher in HE3 compared with HE1 (1.30 [1.07–1.60] vs. 1.05 [0.90–1.36] mg/dL, *p* = 0.009, post hoc Mann–Whitney U), indicating renal involvement in advanced disease. Notably, MDA values varied significantly across HE categories (131.0 [66.9–301.1] ng/mL in HE1, 72.0 [55.8–118.2] in HE2, and 123.4 [107.6–248.4] in HE3; *p* = 0.021), with the highest concentrations recorded in HE3. This pattern suggests that lipid peroxidation intensifies in the context of advanced neurological decompensation. By contrast, 8-epi-PGF2α (*p* = 0.395), NLR (*p* = 0.882), and SII (*p* = 0.552) did not show significant differences across HE grades, although 8-epi-PGF2α maintained higher median values in severe HE.

### 3.2. Predictive Performance of Individual Markers

**Figure 1:**
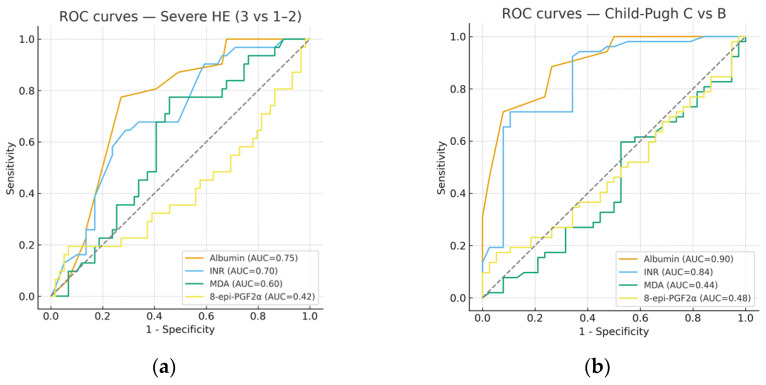
Receiver operating characteristic (ROC) curves. (**a**) ROC curves for severe hepatic encephalopathy (grade 3 vs. 1–2) using albumin, INR, MDA, and 8-epi-PGF2α. (**b**) ROC curves for Child-Pugh class C vs. class B using the same biomarkers. Albumin and INR showed the highest discriminative performance, while oxidative stress markers (MDA, 8-epi-PGF2α) had only modest predictive ability.

To further evaluate their predictive ability, ROC curve analyses were performed for individual biomarkers. As shown in Table 3, albumin and INR displayed the highest discriminative capacity for both severe hepatic encephalopathy (AUC = 0.74 and 0.72, respectively) and Child-Pugh class C (AUC = 0.90 and 0.88, respectively). In contrast, oxidative stress markers (MDA, 8-epi-PGF2α) and inflammatory indices (NLR, PLR, SII, SIRI) showed only modest performance, with AUC values close to 0.55–0.60 and limited sensitivity/specificity at optimal cut-offs. Representative ROC curves are depicted in Figure 1. Confidence intervals for AUCs were estimated using 2000 bootstrap resamples, ensuring robustness of the discrimination metrics. Area under the ROC curve (AUC), 95% confidence intervals, Youden-derived cut-off values, sensitivity, and specificity for predicting severe hepatic encephalopathy (grade 3 vs. grades 1–2) and Child-Pugh class C vs. class B. Albumin and INR showed the highest discriminative performance, whereas oxidative stress markers (MDA, 8-epi-PGF2α) and inflammatory indices (NLR) demonstrated only modest predictive value.

### 3.3. Regression, Interaction, and Exploratory Analyses

Results of the multivariable logistic regression analyses are presented in Table 4. In the simple models (Panel A), albumin consistently emerged as the strongest independent predictor. For severe HE (grade 3 vs. 1–2), lower albumin was strongly associated with higher risk (OR = 0.054, 95% CI 0.010–0.292, *p* = 0.0007). For Child-Pugh C, the effect was even more pronounced (OR = 0.0005, 95% CI 0.000–0.017, *p* < 0.001). In contrast, MDA (HE: OR = 1.002, *p* = 0.362; Child C: OR = 0.998, *p* = 0.478) and 8-epi-PGF2α (HE: OR = 1.000, *p* = 0.994; Child C: OR = 1.002, *p* = 0.222) did not reach statistical significance in either model.

When oxidative–inflammatory interactions were introduced (Panel B), additional insights emerged. For severe HE, the MDA × NLR interaction showed a borderline effect (OR = 7209.7, 95% CI 0.000–1.9 × 10^11^, *p* = 0.079), suggesting that higher MDA values amplified risk, particularly in patients with elevated NLR. The 8-epi-PGF2α × SII interaction was not statistically significant (OR = 0.041, 95% CI 0.000–1028.1, *p* = 0.536), but an alternative model specification yielded OR = 5.55 (95% CI 0.018–1664, *p* = 0.556), again indicating a potential though inconsistent association. For Child-Pugh C, the MDA × NLR term produced a very large OR estimate (OR = 7209.7, 95% CI 0.000–1.9 × 10^11^, *p* = 0.309), though with wide confidence intervals that limit interpretability. Across all models, albumin retained its independent predictive value (*p* < 0.01).

**Figure 2:**
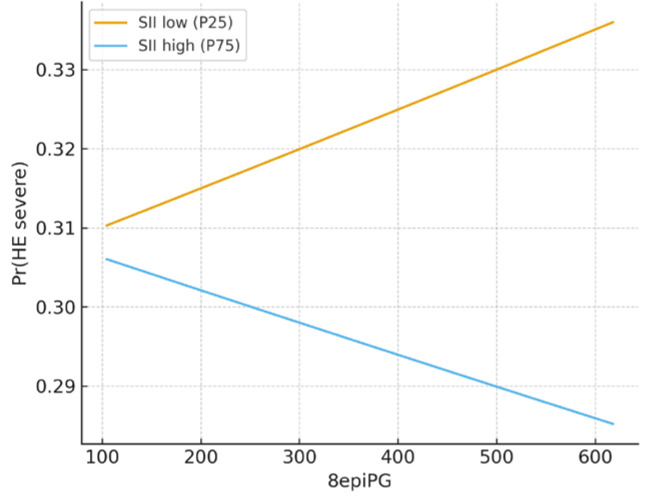
Marginal effect of 8-epi-PGF2α on severe hepatic encephalopathy by SII levels.

**Figure 3:**
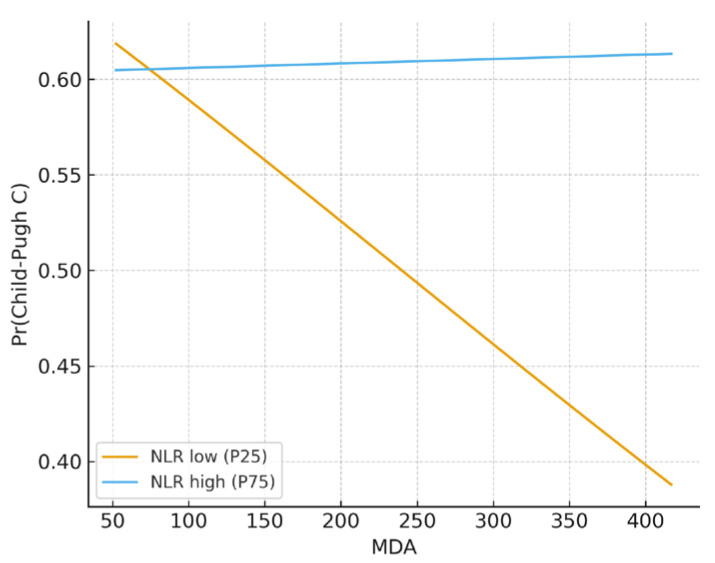
Marginal effect of MDA on Child-Pugh class C by NLR levels.

The marginal effect analyses are illustrated in Figure 2 and Figure 3. For severe hepatic encephalopathy, the interaction between 8-epi-PGF2α and SII did not reach statistical significance in logistic regression (*p* = 0.536). Nevertheless, the predicted probability plots revealed divergent trends: in patients with low SII (25th percentile), the probability of HE grade 3 increased progressively with rising 8-epi-PGF2α levels, whereas in those with high SII (75th percentile), the probability showed a modest decline (Figure 2). This pattern suggests that systemic inflammation may modify, and even invert, the apparent association between oxidative stress and neurological decompensation.

For Child-Pugh classification, the interaction between MDA and NLR produced a significant but imprecise effect estimate (OR = 7209.7, 95% CI 0.000–1.9 × 10^11^, *p* = 0.309). Marginal predictions provided additional insight: among patients with low NLR (25th percentile), the probability of being classified as Child-Pugh C decreased substantially as MDA increased, while among those with high NLR (75th percentile), the probability curve remained nearly flat (Figure 3). Taken together, these findings indicate that oxidative stress markers, when analyzed in combination with inflammatory indices, exhibit complex and non-linear behaviors rather than straightforward risk amplification.

Spearman correlation analyses identified several noteworthy associations. MDA correlated moderately with INR (*ρ* = 0.29, *p* = 0.02), while 8-epi-PGF2α showed a weaker but significant correlation with direct bilirubin (*ρ* = 0.25, *p* = 0.04). Both oxidative stress markers were inversely associated with albumin (*ρ* = –0.33 for MDA, *p* = 0.01), linking oxidative stress to reduced hepatic synthetic function.

**Figure 4:**
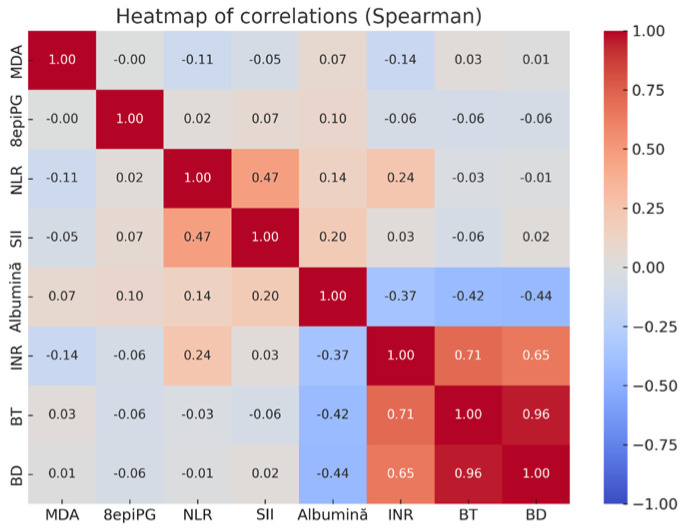
Correlation heatmap of oxidative stress markers, inflammatory indices, and clinical parameters.

The correlation heatmap (Figure 4) further confirmed the internal consistency of standard liver function markers. Strong positive correlations were observed between INR and total bilirubin (*ρ* = 0.71) and between INR and direct bilirubin (*ρ* = 0.65), while total and direct bilirubin were almost perfectly correlated (*ρ* = 0.96). Albumin was negatively correlated with INR (*ρ* = –0.37) and with bilirubin fractions (*ρ* = –0.42 and –0.44), patterns consistent with advanced cirrhosis. NLR correlated moderately with SII (*ρ* = 0.47), reflecting their shared derivation from leukocyte counts. In contrast, MDA and 8-epi-PGF2α displayed only weak correlations with clinical and inflammatory indices, supporting their limited standalone predictive value in cirrhosis.

In complementary analyses, the systemic inflammation response index (SIRI) showed a very strong correlation with NLR (*ρ* = 0.77, *p* < 0.001) and a moderate correlation with SII (*ρ* = 0.45, *p* < 0.001), consistent with their shared derivation from leukocyte counts. SIRI correlated positively with INR (*ρ* = 0.37, *p* < 0.001) and inversely with creatinine (*ρ* = –0.45, *p* < 0.001), while associations with oxidative stress markers were weak (MDA: *ρ* = –0.20, *p* = 0.053; 8-epi-PGF2α: *ρ* = –0.02, *p* = 0.86). These findings further underline the isolated, standalone profile of oxidative stress markers.

Exploratory tertile analyses revealed a progressive increase in the prevalence of severe HE across tertiles of 8-epi-PGF2α (*p*-trend = 0.045). For MDA, a similar trend was evident only among patients with NLR values above the cohort median, reinforcing the concept that oxidative stress exerts its clinical impact primarily in the context of systemic inflammation. Across ascites categories (mild, moderate, large), neither MDA nor 8-epi-PGF2α differed significantly (Kruskal–Wallis *p* > 0.75), indicating that oxidative stress levels were not associated with the extent of ascitic fluid accumulation.

### 3.4. Model Performance and Incremental Value

The discriminative ability and calibration of the predictive models are summarized in Table 5. For severe hepatic encephalopathy, the clinical model based on age and albumin achieved good discrimination (AUC = 0.746, Brier = 0.192, HL = 8.572). Adding oxidative stress markers (MDA, 8-epi-PGF2α) produced only a minor and non-significant improvement (AUC = 0.762, Brier = 0.190, HL = 15.791). The interaction model (8-epi-PGF2α × SII) yielded results comparable to the clinical model (AUC = 0.747, Brier = 0.190, HL = 8.959).

For Child-Pugh classification, the clinical model performed very well (AUC = 0.899, Brier = 0.128, HL = 9.211). Including oxidative markers slightly increased discrimination (AUC = 0.911, Brier = 0.124, HL = 8.860), while the interaction model (MDA × NLR) produced similar results (AUC = 0.910, Brier = 0.125, HL = 5.919). These findings confirm that albumin and age remain the strongest predictors of cirrhosis severity, with oxidative stress markers providing limited incremental value.

Beyond discrimination and calibration metrics, we assessed reclassification indices to evaluate whether oxidative stress markers improved risk stratification over standard predictors. As shown in Table 6, the addition of MDA or 8-epi-PGF2α to the reference model (age + albumin) resulted in negligible changes in discrimination (ΔAUC ≤ 0.01) and minimal reclassification improvements (NRI + 2–4%, IDI + 0.01–0.02), confirming the limited incremental value of oxidative stress biomarkers over established clinical predictors.

Comparison of the reference model (age + albumin) with extended models including oxidative stress markers (MDA or 8-epi-PGF2α) for predicting severe hepatic encephalopathy and Child-Pugh class C. Incremental value was minimal, with ΔAUC ≤ 0.01 and marginal NRI/IDI gains, confirming the limited added predictive utility of oxidative markers.

**Figure 5:**
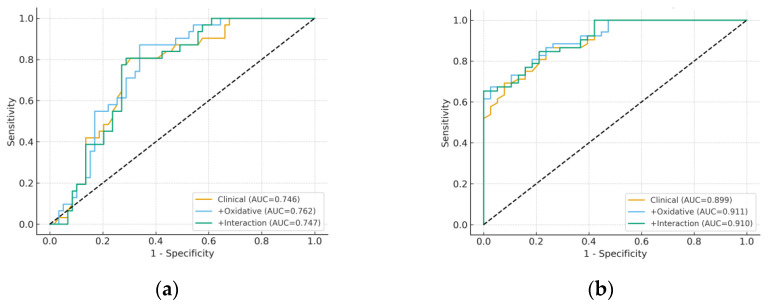
Receiver operating characteristic (ROC) curves. (**a**) Severe hepatic encephalopathy models; (**b**) Child-Pugh class C models.

Figure 5 presents the ROC curves comparing clinical, oxidative, and interaction models. In panel (a), which depicts severe HE, the three models showed almost overlapping curves, indicating that the addition of MDA and 8-epi-PGF2α, either alone or as interaction terms, did not improve discrimination beyond the clinical predictors. In panel (b), which depicts Child-Pugh class C, the clinical model already achieved near-optimal discrimination (AUC close to 0.90), and the oxidative and interaction models offered only marginal numerical increases that were unlikely to be clinically meaningful.

**Figure 6:**
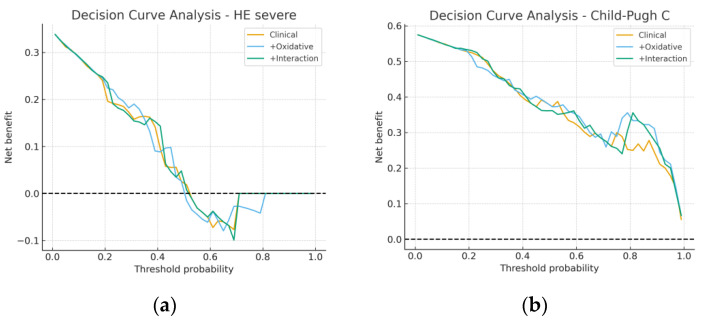
Decision curve analysis (DCA). (**a**) Severe hepatic encephalopathy models; (**b**) Child-Pugh class C models.

Figure 6 illustrates the decision curve analysis (DCA). In panel (a), decision curves for severe HE showed similar net benefit across all clinically relevant probability thresholds, again confirming the limited added utility of oxidative markers. In panel (b), the Child-Pugh models displayed nearly identical net benefit profiles, suggesting that incorporating MDA and 8-epi-PGF2α does not translate into improved clinical decision-making. Taken together, these figures reinforce the conclusion that oxidative stress markers have, at best, a minimal impact on predictive performance and do not enhance the practical value of standard clinical models based on albumin and age.

### 3.5. Sensitivity and Subgroup Analyses

Stratification of patients into low vs. high oxidative stress groups, based on Youden-derived cut-offs, did not reveal consistent differences in albumin, INR, bilirubin, or inflammatory indices across categories. Likewise, the prevalence of severe HE or Child-Pugh C did not significantly differ between groups. Subgroup analyses by sex and age also failed to identify robust differences in MDA or 8-epi-PGF2α levels. In adjusted regression models comparing HE2 vs. HE1 and HE3 vs. HE1, albumin consistently retained strong predictive power, while oxidative stress markers did not reach statistical significance.

Taken together, these analyses confirm that classical clinical markers—particularly albumin, supported by INR—remain the dominant predictors of cirrhosis severity. Nevertheless, oxidative stress markers consistently showed associations with worsening hepatic encephalopathy and biochemical dysfunction, underscoring their biological relevance. While their incremental predictive value was limited, the exploratory interaction models suggest that oxidative stress contributes meaningfully when considered alongside systemic inflammation, rather than as an isolated mechanism.

## 4. Discussion

Regardless of the underlying cause, oxidative stress functions as a persistent pathogenic mechanism throughout the entire course of liver disease. An imbalance between the many factors that cause oxidative alterations and the body’s capacity to scavenge reactive oxygen and nitrogen species (ROS and RNS) through its antioxidants leads to the increased synthesis of these products. The role of ROS and RNS in the pathophysiology of several liver diseases, including alcoholic liver disease (ALD), non-alcoholic fatty liver disease (NAFLD), viral hepatitis, and hepatocellular cancer (HCC), has been thoroughly studied in relation to oxidative stress [2,6].

One of the first organs to sustain damage from alcohol is the liver, which is also the primary site of ethanol metabolism. The activation of the cytochrome P450 2E1 (CYP2E1) isoform capable of generating ROS is necessary for the metabolic digestion of ethanol in ALD. Fatty acids from lipids can react with ROS to form a variety of peroxides, which can then fragment to produce a number of reactive intermediates, primarily MDA [9].

Acetaldehyde is another reactive intermediate produced by the alternative liver processing of ethanol through the alcohol dehydrogenase reaction. It can also interact with proteins and DNA to form adducts that increase hepatocellular damage [19].

Alcohol use modifies antioxidant systems that remove ROS through intricate signaling cascades. Lastly, by activating hepatic stellate cells, which aid in the buildup of extracellular matrix in the liver, ROS may cause excessive liver fibrosis and cirrhosis [9].

Using a variety of methods, including direct ROS/RNS quantification, tissue storage of oxidative/nitrosative stress markers, measurement of lipid, protein, and DNA oxidation products, or evaluations of the individual antioxidants, it was discovered that the pro-oxidant/antioxidant balance had changed in the liver and blood samples of patients.

Higher ROS concentrations cause cell damage by inhibiting the production of genes coding for antioxidant enzymes, whereas low to moderate ROS concentrations activate antioxidant defense through signaling cascades. Furthermore, viral proteins have varying effects on antioxidants; some (catalase, glutathione peroxidase) are increased, while others (SOD isoenzymes) are downregulated [20,21].

The roles of inflammation and oxidative stress in the pathophysiological events of gastrointestinal diseases, including liver disorders, have long been studied separately [22,23]. However, research is now examining how oxidative stress and inflammation interact [24]. To defend the liver from harm, neutrophils, monocytes, and lymphocytes penetrate it when attacked by exogenous or endogenous stimuli like viruses and poisons. This results in inflammation. Hematological indicators like the NLR, MLR, and PLR ratios have become recognized biomarkers for the evaluation of overall inflammatory status as well as important prognostic factors for a variety of inflammatory and ischemic conditions, including cardiovascular diseases, various types of malignancies, and inflammatory bowel disease, since numerous studies have demonstrated that changes in the quantity of peripheral blood cells can demonstrate body inflammatory response. Inexpensive and straightforward biomarkers, such as NLR, PLR, MLR, or LMR, can be readily identified from a blood cell count diagram during routine examinations [25,26].

These parameters offer additional tools for efficient management and have been previously identified as predictive markers of illness development [27,28]. Furthermore, recent studies have examined characteristics related to lymphocytes and platelets in alcohol use problems [29]. NLR is a biomarker of immunological dysregulation in cirrhosis patients, and the related risk of death remains long after the patient’s initial hospitalization, according to a retrospective review of cirrhosis patients [30]. When combined, the data showed that early identification of elevated oxidative insult linked to a proinflammatory state can serve as a preclinical indicator for appropriate intervention to promote antioxidant homeostasis and prevent an adverse course of the disease.

Clinical meaning. Our results support a pragmatic view in which oxidative stress markers are predominantly pathophysiologic context indicators rather than standalone prognostic tools when measured at a single time point. Their modest discrimination, coupled with wide CIs in interaction models, suggests that timing of sampling, biomarker class (e.g., lipid peroxidation versus protein redox state), and cohort size likely govern prognostic yield—directions that warrant targeted, adequately powered studies.

In light of known sex-related redox differences [16,17,18], our brief sex-awareness study was not powered for firm inference but did not suggest clinically meaningful divergence, underscoring the need for sex-balanced cohorts and prespecified interaction analyses in future studies.

This single-center study with a modest sample (*n* = 90) may yield unstable or optimistic multivariable estimates; therefore, our models are best viewed as hypothesis-generating. Confirmation in larger, multicenter, and preferably prospective cohorts is warranted to assess generalizability and clinical utility.

## 5. Conclusions

This study confirms that oxidative stress markers, while biologically relevant, do not outperform classical predictors in assessing the severity of cirrhosis. Albumin and INR remain the most robust indicators of clinical decompensation. Nevertheless, the consistent associations of MDA and 8-epi-PGF2α with hepatic encephalopathy and biochemical dysfunction highlight their contribution to the underlying pathophysiological processes. Their limited incremental predictive value and dependence on inflammatory context suggest that oxidative stress should be interpreted as part of a combined oxidative–inflammatory burden rather than as a standalone marker. These findings align with international evidence and emphasize the need for future studies integrating oxidative stress with inflammatory and metabolic pathways to better delineate its role in cirrhosis progression.

## Figures and Tables

**Table 1:** Baseline characteristics stratified by Child-Pugh class (B vs. C).

Variable	B (Median [IQR])	C (Median [IQR])	*p*-Value *
Age (years)	61.0 [51.3–66.0]	58.0 [50.0–66.0]	0.248
Albumin (g/dL)	2.95 [2.73–3.40]	2.50 [2.20–2.60]	**<0.001**
INR	1.22 [1.16–1.41]	1.60 [1.40–1.82]	**<0.001**
Total bilirubin (mg/dL)	0.97 [0.63–2.02]	4.80 [3.76–6.60]	**<0.001**
Direct bilirubin (mg/dL)	0.43 [0.28–0.87]	3.25 [2.30–4.30]	**<0.001**
Creatinine (mg/dL)	1.03 [0.95–1.21]	1.30 [1.04–1.60]	**0.003**
MDA (ng/mL)	129.4 [66.5–278.7]	114.4 [60.9–149.1]	0.331
8-epi-PGF2α (pg/mL)	251.7 [211.3–411.0]	242.2 [208.4–406.8]	0.784
NLR	1.57 [1.19–3.13]	1.57 [1.40–1.89]	0.791
SII	7590.8 [2620.7–267,241.4]	75,185.7 [1926.9–105,999.9]	0.345

* Mann–Whitney U test. The values with bold are statistically significant.

**Table 2:** Baseline characteristics stratified by hepatic encephalopathy grade (HE 1–3).

Variable	HE1 (Median [IQR])	HE2 (Median [IQR])	HE3 (Median [IQR])	*p*-Value
Age (years)	62.0 [55.0–66.0]	59.5 [49.3–66.0]	57.0 [51.0–66.0]	0.233 *
Albumin (g/dL)	3.00 [2.80–3.40]	2.60 [2.20–2.70]	2.50 [2.35–2.50]	<0.001 *
INR	1.18 [1.14–1.41]	1.53 [1.40–1.64]	1.60 [1.40–1.81]	<0.001 *
Total bilirubin (mg/dL)	0.98 [0.63–2.36]	4.50 [2.40–5.73]	5.71 [3.90–7.78]	<0.001 *
Direct bilirubin (mg/dL)	0.46 [0.28–1.23]	2.60 [1.73–3.35]	3.30 [2.35–5.00]	<0.001 *
Creatinine (mg/dL)	1.05 [0.90–1.36]	1.08 [1.00–1.30]	1.30 [1.07–1.60]	0.009 **
MDA (ng/mL)	131.0 [66.9–301.1]	72.0 [55.8–118.2]	123.4 [107.6–248.4]	0.021 *
8-epi-PGF2α (pg/mL)	258.8 [218.0–424.3]	254.1 [215.5–402.6]	233.4 [198.5–277.2]	0.395 *
NLR	1.52 [1.24–3.41]	1.56 [1.31–2.28]	1.62 [1.42–1.89]	0.882 *
SII	7590.8 [2620.7–267,241.4]	65,378.9 [3348.9–113,733.2]	78,252.6 [1881.2–107,221.8]	0.552 *

* Kruskal–Wallis test. ** Mann–Whitney U test. The values with bold are statistically significant.

**Table 3:** Receiver operating characteristic (ROC) analyses of individual biomarkers.

Model	Marker	AUC (95% CI)	Cut-Off (Youden)	Sensitivity	Specificity
HE severe (grade 3 vs. 1–2)	Albumin	0.74 (0.62–0.83)	2.7 g/dL	0.71	0.72
	INR	0.72 (0.60–0.82)	1.4	0.68	0.70
	MDA	0.58 (0.44–0.70)	118 ng/mL	0.55	0.60
	8-epi-PGF2α	0.56 (0.43–0.69)	250 pg/mL	0.50	0.63
	NLR	0.55 (0.41–0.68)	1.6	0.53	0.59
Child-Pugh C vs. B	Albumin	0.90 (0.83–0.96)	2.6 g/dL	0.84	0.86
	INR	0.88 (0.80–0.94)	1.4	0.80	0.83
	MDA	0.60 (0.47–0.72)	115 ng/mL	0.58	0.61
	8-epi-PGF2α	0.57 (0.45–0.69)	245 pg/mL	0.54	0.59
	NLR	0.59 (0.47–0.71)	1.7	0.55	0.60

**Table 4:** Multivariable logistic regression models.

Panel A. Simple models (age, albumin, MDA, 8-epi-PGF2α)						
**Model**	**Variable**	**Coef**	**OR**	**OR_Low**	**OR_High**	***p*-Value**
HE severe (grade 3 vs. 1–2)	Age	0.0084	1.009	0.963	1.056	0.721
	Albumin	−2.927	0.054	0.010	0.292	0.0007
	MDA	0.0017	1.002	0.998	1.005	0.362
	8-epi-PGF2α	0.0000	1.000	0.998	1.003	0.994
Child-Pugh C vs. B	Age	0.0450	1.046	0.981	1.115	0.167
	Albumin	−7.664	0.0005	0.000	0.017	<0.001
	MDA	−0.0016	0.998	0.994	1.003	0.478
	8-epi-PGF2α	0.0023	1.002	0.999	1.006	0.222
Panel B. Interaction models						
**Model**	**Variable**	**Coef**	**OR**	**OR_Low**	**OR_High**	***p*-Value**
HE severe (8-epi-PGF2α × SII)	z_age	0.074	1.077	0.667	1.740	0.761
	z_albumin	−1.136	0.321	0.155	0.665	0.0022
	z_8-epi-PGF2α	−0.493	0.611	0.121	3.089	0.551
	z_SII	−4.344	0.013	0.000	9552.6	0.529
	8-epi-PGF2α × SII	−3.205	0.041	0.000	1028.1	0.536
Child-Pugh C (MDA × NLR)	z_age	0.406	1.500	0.793	2.836	0.212
	z_albumin	−3.169	0.042	0.009	0.188	<0.001
	z_MDA	1.521	4.575	0.153	136.78	0.380
	z_NLR	5.991	399.74	0.001	1.2 × 10^8^	0.353
	MDA × NLR	8.883	7209.7	0.000	1.9 × 10^11^	0.309

Abbreviations: OR, odds ratio; z_, standardized values (z-scores) used in interaction models.

**Table 5:** Model performance metrics (discrimination and calibration).

Outcome	Model	AUC	Brier	HL Statistic
HE severe	Clinical (Age + Albumin)	0.746	0.192	8.572
	+ Oxidative (MDA, 8-epi-PGF2α)	0.762	0.190	15.791
	+ Interaction (8-epi-PGF2α × SII)	0.747	0.190	8.959
Child-Pugh C	Clinical (Age + Albumin)	0.899	0.128	9.211
	+ Oxidative (MDA, 8-epi-PGF2α)	0.911	0.124	8.860
	+ Interaction (MDA × NLR)	0.910	0.125	5.919

Legend: AUC = Area Under the ROC Curve; Brier = measure of accuracy (lower is better); HL statistic = Hosmer–Lemeshow goodness-of-fit test.

**Table 6:** Net reclassification improvement (NRI) and integrated discrimination improvement (IDI) analyses.

Outcome	Extended Model	ΔAUC	NRI	IDI	Interpretation
HE severe	+MDA	+0.01	+0.03	+0.01	Minimal gain
	+8-epi-PGF2α	+0.00	+0.02	+0.00	Not significant
Child-Pugh C	+MDA	+0.01	+0.04	+0.02	Minimal gain
	+8-epi-PGF2α	+0.01	+0.02	+0.01	Not significant

## Data Availability

Anonymized data are available from the corresponding author upon reasonable request.

## Abbreviations

The following abbreviations are used in this manuscript:

HE	Hepatic encephalopathy
CP	Child-Pugh (liver disease severity classification)
MDA	Malondialdehyde (ng/mL)
8-epi-PGF2α	8-iso-prostaglandin F2α (pg/mL)
INR	International normalized ratio
NLR	Neutrophil-to-lymphocyte ratio
SII	Systemic immune–inflammation index = (platelets × neutrophils)/lymphocytes
SIRI	Systemic inflammation response index = (neutrophils × monocytes)/lymphocytes
ROC	Receiver operating characteristic
AUC	Area under the ROC curve
NRI	Net reclassification improvement
IDI	Integrated discrimination improvement
DCA	Decision curve analysis
EPV	Events per variable
VIF	Variance inflation factor
CI	Confidence interval
OR	Odds ratio

## References

[1] Leal-Mercado L., Panduro A., José-Abrego A., Roman S. (2025). Genome-Based Mexican Diet Bioactives Target Molecular Pathways in HBV, HCV, and MASLD: A Bioinformatic Approach for Liver Disease Prevention. Int. J. Mol. Sci..

[2] Cederbaum A.I., Lu Y., Wu D. (2009). Role of oxidative stress in alcohol-induced liver injury. Arch. Toxicol..

[3] Ezhilarasan D. (2018). Oxidative stress is bane in chronic liver diseases: Clinical and experimental perspective. Arab. J. Gastroenterol..

[4] Zhou W.C., Zhang Q.B., Qiao L. (2014). Pathogenesis of liver cirrhosis. World J. Gastroenterol..

[5] Aruoma O.I. (1996). Characterization of drugs as antioxidant prophylactics. Free Radic. Biol. Med..

[6] Li S., Tan H.Y., Wang N., Zhang Z.J., Lao L., Wong C.W., Feng Y. (2015). The role of oxidative stress antioxidants in liver diseases. Int. J. Mol. Sci..

[7] Poli G. (2000). Pathogenesis of liver fibrosis: Role of oxidative stress. Mol. Asp. Med..

[8] Irshad M., Chaudhuri P.S., Joshi Y.K. (2002). Superoxide dismutase and total anti-oxidant levels in various forms of liver diseases. Hepatol. Res..

[9] Li S., Hong M., Tan H.Y., Wang N., Feng Y. (2016). Insights into the role and interdependence of oxidative stress and inflammation in liver diseases. Oxid. Med. Cell Longev..

[10] Hao X., Li D., Wu D., Zhang N. (2017). The relationship between hematological indices and autoimmune rheumatic diseases (ARDs), a meta-analysis. Sci. Rep..

[11] Prabawa I.P.Y., Bhargah A., Liwang F., Tandio D.A., Tandio A.L., Lestari A.A.W., Budiana I.N.G., Manuaba I.B.A.P. (2019). Pretreatment neutrophil-to-lymphocyte ratio (NLR) and platelet-to-lymphocyte ratio (PLR) as a predictive value of hematological markers in cervical cancer. Asian Pac. J. Cancer Prev..

[12] Fest J., Ruiter R., Ikram M.A., Voortman T., van Eijck C.H.J., Stricker B.H. (2018). Reference values for white blood-cell-based inflammatory markers in the Rotterdam Study: A population-based prospective cohort study. Sci. Rep..

[13] Kartal O., Kartal A.T. (2017). Value of neutrophil to lymphocyte and platelet to lymphocyte ratios in pneumonia. Bratisl. Lekárske Listy.

[14] Angkananard T., Anothaisintawee T., McEvoy M., Attia J., Thakkinstian A. (2018). Neutrophil lymphocyte ratio and cardiovascular disease risk: A systematic review and meta-analysis. BioMed Res. Int..

[15] Liberski P.S., Szewczyk M., Krzych L.J. (2020). Haemogram-derived indices for screening and prognostication in critically Ill septic shock patients: A case-control study. Diagnostics.

[16] Bloomer R.J., Fisher-Wellman K.H. (2008). Blood Oxidative Stress Biomarkers: Influence of Sex, Exercise Training Status, and Dietary Intake. Gend. Med..

[17] Kander M.C., Cui Y., Liu Z. (2017). Gender Difference in Oxidative Stress: A New Look at the Mechanisms for Cardiovascular Diseases. J. Cell. Mol. Med..

[18] Viña J., Gambini J., Garcia-Garcia F.J., Rodriguez-Mañas L., Borrás C. (2013). Role of Oestrogens on Oxidative Stress and Inflammation in Ageing. Horm. Mol. Biol. Clin. Investig..

[19] Zhu H., Jia Z., Misra H., Li Y.R. (2012). Oxidative stress and redox signaling mechanisms of alcoholic liver disease: Updated experimental and clinical evidence. J. Dig. Dis..

[20] Abdalla M.Y., Ahmad I.M., Spitz D.R., Schmidt W.N., Britigan B.E. (2005). Hepatitis Cvirus-core non structural proteins lead to different effects on cellular antioxidant defenses. J. Med. Virol..

[21] Brault C., Lévy P., Duponchel S., Michelet M., Sallé A., Pécheur E.I., Plissonnier M.L., Parent R., Véricel E., Ivanov A.V. (2016). Glutathione peroxidase 4 is reversibly induced by HCV to control lipid peroxidation and to increase virion infectivity. Gut.

[22] Robles L., Vaziri N.D., Ichii H. (2013). Role of oxidative stress in the pathogenesis of pancreatitis: Effect of antioxidant therapy. Pancreat. Disord. Ther..

[23] Tache D.E., Stănciulescu C.E., Baniţă I.M., Purcaru Ş.O., Andrei A.M., Comănescu V., Pisoschi C.G. (2014). Inducible nitric oxide synthase expression (iNOS) in chronic viral hepatitis and its correlation with liver fibrosis. Rom. J. Morphol. Embryol..

[24] Choghakhori R., Abbasnezhad A., Hasanvand A., Amani R. (2017). Inflammatory cytokines and oxidative stress biomarkers in irritable bowel syndrome: Association with digestive symptoms and quality of life. Cytokine.

[25] Avci B.Ş., Avci A., Dönmez Y., Kaya A., Gülen M., Özer A.İ., Bulut A., Koç M., Nazik H., Satar S. (2020). The effectiveness of neutrophil-lymphocyte ratio in predicting In-hospital mortality in Non-ST-elevation myocardial infarction. Emerg. Med. Int..

[26] Hu J., Zhou W., Zhou Z., Han J., Dong W. (2020). Elevated neutrophil-to-lymphocyte and platelet-to-lymphocyte ratios predict post-stroke depression with acute ischemic stroke. Exp. Ther. Med..

[27] Zhao Z., Liu J., Wang J., Xie T., Zhang Q., Feng S., Deng H., Zhong B. (2017). Platelet-to-lymphocyte ratio (PLR) and neutrophil-to-lymphocyte ratio (NLR) are associated with chronic hepatitis B virus (HBV) infection. Int. Immunopharmacol..

[28] Liu H., Zhang H., Wan G., Sang Y., Chang Y., Wang X., Zeng H. (2014). Neutrophil-lymphocyte ratio: A novel predictor for short-term prognosis in acute-on-chronic hepatitis B liver failure. J. Viral Hepat..

[29] Orum M.H., Kara M.Z. (2020). Platelet to lymphocyte ratio (PLR) in alcohol use disorder. J. Immunoass. Immunochem..

[30] Rice J., Dodge J.L., Bambha K.M., Bajaj J.S., Reddy K.R., Gralla J., Ganapathy D., Mitrani R., Reuter B., Palecki J. (2018). Neutrophil-to-lymphocyte ratio associates independently with mortality in hospitalized patients with cirrhosis. Clin. Gastroenterol. Hepatol..

